# Mitochondrial Dynamics Regulation in Skin Fibroblasts from Mitochondrial Disease Patients

**DOI:** 10.3390/biom10030450

**Published:** 2020-03-13

**Authors:** Takeshi Tokuyama, Asei Hirai, Isshin Shiiba, Naoki Ito, Keigo Matsuno, Keisuke Takeda, Kanata Saito, Koki Mii, Nobuko Matsushita, Toshifumi Fukuda, Ryoko Inatome, Shigeru Yanagi

**Affiliations:** Laboratory of Molecular Biochemistry, School of Life Sciences, Tokyo University of Pharmacy and Life Sciences, Hachioji, Tokyo 192-0392, Japan; tokuyama@toyaku.ac.jp (T.T.); s148060@toyaku.ac.jp (A.H.); s139053@toyaku.ac.jp (I.S.); s139010@toyaku.ac.jp (N.I.); s116212@toyaku.ac.jp (K.M.); stakeda@toyaku.ac.jp (K.T.); s159047@toyaku.ac.jp (K.S.); s159101@toyaku.ac.jp (K.M.); matsun@toyaku.ac.jp (N.M.); tfukuda@toyaku.ac.jp (T.F.); inatome@ls.toyaku.ac.jp (R.I.)

**Keywords:** mitochondrial disease, mitochondrial fragmentation, Drp1

## Abstract

Mitochondria are highly dynamic organelles that constantly fuse, divide, and move, and their function is regulated and maintained by their morphologic changes. Mitochondrial disease (MD) comprises a group of disorders involving mitochondrial dysfunction. However, it is not clear whether changes in mitochondrial morphology are related to MD. In this study, we examined mitochondrial morphology in fibroblasts from patients with MD (mitochondrial myopathy, encephalopathy, lactic acidosis, and stroke-like episodes (MELAS) and Leigh syndrome). We observed that MD fibroblasts exhibited significant mitochondrial fragmentation by upregulation of Drp1, which is responsible for mitochondrial fission. Interestingly, the inhibition of mitochondrial fragmentation by Drp1 knockdown enhanced cellular toxicity and led to cell death in MD fibroblasts. These results suggest that mitochondrial fission plays a critical role in the attenuation of mitochondrial damage in MD fibroblasts.

## 1. Introduction

Mitochondrial disease (MD) comprises a group of disorders involving mitochondrial dysfunction. MD is caused by defects in the mitochondrial DNA-encoded genes or nuclear genes that regulate mitochondrial function. Mitochondrial myopathy, encephalopathy, lactic acidosis, and stroke-like episodes (MELAS) is a syndrome caused by mitochondrial DNA mutations. The main cause of pathology in MELAS is a deficiency of taurine modification at the wobble uridine of the mutant transfer RNA leucine (UUR), causing UUG codon-specific translational failure and defective synthesis of mitochondrial protein [1,2]. MELAS is caused by at least 32 mutations in the mitochondrial DNA that control different mitochondrial genes [3]. The most common variant, accounting for more than 80% of MELAS cases, is the mitochondrial m.3243A>G variant [4,5]. Leigh syndrome is a group of progressive neurodegenerative disorders that typically affect infants and can be caused by mutations in mitochondrial or nuclear DNA. Leigh syndrome is a highly genetically heterogeneous MD. Mutations can be heteroplasmic as a result of the presence of multiple mitochondrial DNA (mtDNA) molecules in individual mitochondria; this means that mutated mtDNA and normal mtDNA coexist in mitochondria [6]. It has been suggested that Leigh syndrome may be caused by mutations in more than 35 different genes from the nucleus and mitochondria that are involved in all of the respiratory chain complexes [7]. However, in multiple cases, the exact genetic cause of Leigh syndrome remains unknown [8]. Currently, there is no cure or effective treatment for MD, but recent research has shown the potential benefits of some approaches, at least in preclinical in vivo models [9,10,11]. Many researchers assume that both bulk and mitochondrial autophagy play protective roles in MD because the accumulation of damaged mitochondria and other toxic aggregates causes deterioration of the pathophysiology of the cell [12]. Preclinical studies suggest that a potential therapeutic target of disease progression is the mammalian target of rapamycin (mTOR) pathway, a biological route fundamental for regulating cell metabolism and physiology [13]. In mice models of Leigh syndrome, treatment with rapamycin, an mTOR inhibitor, extended lifespan and reduced disease progression and severity [14]. Civiletto et al. provided evidence that rapamycin induces improvements in mitochondrial function and ultrastructure, indicating powerful clearance of dysfunctional organelles via activation of autophagic flux in skeletal muscle.

Mitochondria are highly dynamic organelles that continuously fuse, divide, and move, and mitochondrial function is controlled and maintained by these morphologic changes. Mitochondrial fission is specifically mediated by dynamin-related guanosine triphosphatase (GTPase) protein 1 (Drp1); in addition, dynamin-related GTPases mitofusin (Mfn) and optic atrophy 1 (OPA1) are associated with the outer and inner mitochondrial membranes, respectively, and mediate fusion of these membranes [15,16,17,18,19]. The most direct consequence of mitochondrial division and fusion is the change in size of the mitochondria [20,21,22]. Mitochondrial fission via Drp1 has been thought to regulate mitophagy by dividing mitochondria into fragments suitable for autophagosome engulfment [23,24,25] and/or separating damaged subdomains of mitochondria for elimination [26]. Mitochondrial fusion enables efficient mixing of mitochondrial contents and produces an expanded mitochondrial network. Both effects are beneficial under conditions of high energy demand, and disruption of mitochondrial fusion results in mitochondrial dysfunction and loss of respiratory capacity [27,28,29]. Although mitochondrial dysfunction is considered to be one of the causes of MD, it is unknown whether changes in mitochondrial morphology are related to MD.

In this study, we examined mitochondrial morphology in fibroblasts from two patients with MD. We observed that MD fibroblasts exhibited significant mitochondrial fragmentation by upregulation of Drp1, which controls mitochondrial fission. Moreover, restraining mitochondrial fission enhanced cellular toxicity and cell death in MD fibroblasts, suggesting that mitochondrial fission plays a critical role in the attenuation of mitochondrial damage in MD fibroblasts.

## 2. Materials and Methods

### 2.1. Cell Culture and Transfection

Primary skin fibroblasts from two mitochondrial disease patients and age-matched healthy controls were cultured in a fibroblast growth medium (FGM; Lonza) constituting fibroblast basal medium (FBM; Lonza) supplemented with gentamicin/amphotericin B (antibiotic/antifungal) and growth factors (rhFGF-B, insulin, fetal bovine serum; all from BulletKits®, Lonza Cat. No. CC-3132 FGM™-2 BulletKit™). Cells were transfected with RNAiMax (Invitrogen) according to the manufacturer’s protocol. The small interfering RNA (siRNA) used was siDrp1, as described previously [30].

### 2.2. Antibodies and Reagents

Anti α-tubulin was purchased from Sigma. Anti-LC3, anti-cleaved caspase-3, and anti-VDAC were from Cell Signaling Technology. Anti-p62 was from MBL. Anti-HSP60 was from Enzo Life Sciences. Anti-Drp1 was from Abcam. Anti-Tom20, anti-MID49, anti-mitofusin1, anti-Fis1, and anti-MFF were from Proteintech. Anti-OPA1 was from BD Bioscience. Anti-mitofusin2 was from Santa Cruz Biotechnology. BafilomycinA1 was from LC Laboratories.

### 2.3. Immunoblotting

Whole-cell lysates were separated by SDS-PAGE and transferred to polyvinylidene fluoride (PVDF) membranes (Millipore). The blots were probed with the indicated antibodies, and protein bands on the blots were visualized by the enhanced chemiluminescence reagent (Millipore). Band intensity was measured using ImageJ.

### 2.4. Measurement of Cellular Oxygen Consumption and Extracellular Acidification

The cellular oxygen consumption rate (OCR) and extracellular acidification rate (ECAR) were detected by the XF Cell Mito Stress Test™ using an XFp Extracellular Flux Analyzer™ (Agilent Technologies). It was performed according to the manufacturer’s protocol.

### 2.5. Morphological Analysis by Immunofluorescence Microscopy 

Cells were fixed with 4% paraformaldehyde in phosphate-buffered saline (PBS) for 1 h at room temperature, then washed twice with PBS, permeabilized with 0.2% Triton X-100 in PBS for 5 min, then washed four times with PBS, and blocked with 1% bovine serum albumin in PBS, all at room temperature. For double staining, the cells were incubated with appropriate primary Abs for 1 h at room temperature, washed three times with PBS, and then incubated with appropriate secondary Abs for 30 min. The samples were washed as described above, mounted using a fluorescent mounting medium (Dako), and analyzed using a Keyence BZ-9000 confocal fluorescence microscope. The quantification of mitochondrial morphology was performed in a blinded manner.

### 2.6. Flow Cytometry

Annexin V-FITC staining was performed with an Annexin V-FITC apoptosis detection kit (BioVision) according to the manufacturer’s protocol. For measurement of intracellular oxidative stress, cells were incubated with 5 µM CellROX® Green Reagent for 30 min at 37 °C and then washed with PBS. For measurement of mitochondrial oxidative stress, cells were incubated with 2.5 µM MitoSOX Red Reagent for 30 min at 37 °C and then washed with PBS. For detection of mitochondrial membrane potential, cells were incubated with 0.5 µM 3,3′-dihexyloxacarbocyanine iodide (DiOC6) for 20 min at 37 °C and then washed with PBS. Fluorescence was analyzed by a flow cytometer (Sony SH800).

### 2.7. ATP Assay

A CellTiter-Glo™ luminescent cell viability assay kit (Promega) was used to evaluate the intracellular adenosine triphosphate (ATP) content. Briefly, cells were seeded in 96-well plates (1000 cells/well) and incubated for 12 h. The CellTiter-Glo reagent (50 μL) was then added directly into each well and incubated for 10 min prior to reading the plate using an EnSpire™ Multimode Plate Reader (PerkinElmer).

### 2.8. RNA Isolation and qRT-PCR

Total RNA was isolated from mammalian cells using a RNeasy kit (Qiagen) and subjected to reverse transcription to cDNA using the ReverTra Ace qPCR RT kit (Toyobo), following the manufacturer’s protocol. PCR was performed using THUNDERBIRD SYBR qPCR Mix (Toyobo). The PCR conditions were as follows: 95 °C for 10 min, followed by 40 cycles at 95 °C for 15 s, 60 °C for 1 min. RT-PCR was performed using miScript SYBR Green PCR (Qiagen). The following primers were used: Drp1: Forward, 5′-AGGTTGC¬CCGTGACAAATGA-3′, reverse, 5′-ATCAGCAAAGTCGGGGTGTT-3′; β-actin: Forward, 5′-AGAGCTACGAGCTGCCTGAC-3′, reverse, 5′-AGCACTGTGTTGGCGTACAG-3′.

### 2.9. Heteroplasmy Determination by RFLP

For restriction fragment length polymorphism (RFLP) analysis, the mtDNA 3243 locus was amplified using the following primers: Forward, 5′-ACCTCGGAGCAGAACCCAACCTC-3′; reverse, 5′-CAGCGAAGGGTTGTAGTAGCCCGTAG-3′, which produced a PCR product of 641 bp. In the presence of the A3243G mutation, the PCR product was digested by ApaI into two fragments of 426 and 215 bp. The cycling protocol used was as follows: Initial denaturation at 94 °C for 1 min, 40 cycles of denaturation at 98 °C for 10 s, annealing at 62 °C for 15 s, and elongation at 68 °C for 8 s. The final cycle was followed by extension at 68 °C for 30 s. The digested PCR products were separated on a 2% agarose gel and stained with ethidium bromide. For RFLP analysis, the mtDNA 10158 locus was amplified using the following primers: Forward, 5′-GCCGCCGCCTGATACTGGCATTTTG-3′; reverse, 5′-TATAGGGTCGAAGCCGCACTCGTAAGGGGTCG-3′. The amplified 268 bp fragment was digested with TaqI to produce 246 + 22 bp fragments from the wildtype sequence, whereas the mutant sequence remained undigested. The cycling protocol used was as follows: Initial denaturation at 94 °C for 4 min, 45 cycles of denaturation at 98 °C for 10 s, annealing at 56 °C for 15 s, and elongation at 68 °C for 10 s. The final cycle was followed by extension at 68 °C for 5 min. These fragments were separated electrophoretically through a 4% low-melting agarose gel after ethidium bromide staining. ImageJ software (version 1.51m9; NIH, Bethesda, MD, USA) was used to analyze the signal intensity of the bands.

### 2.10. Statistical Analysis

Statistical analysis was performed with GraphPad Prism version 8 (GraphPad Software, Inc.). All results are expressed as the mean ± SD. Obtained data were compared between independent experiments using two-tailed Student’s t-test. The number of independent experiments is shown as n. Comparisons between multiple groups were assessed by one-way ANOVA with Dunnett’s post hoc analysis. *P < 0.05; **P < 0.01; NS: Not significant.

## 3. Results

### 3.1. Characterization in MD Fibroblasts

**Figure 1:**
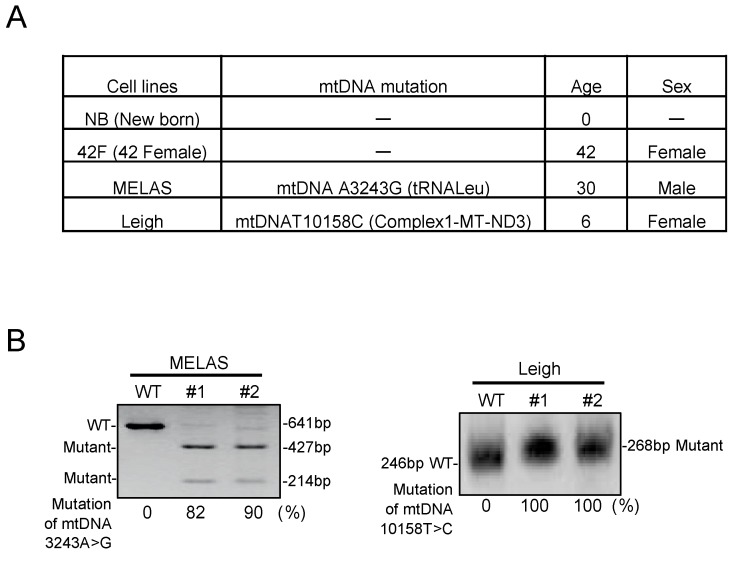
Isolation and characterization of fibroblasts from mitochondrial disease patients. (**A**) Overview of cell lines used in this study. (**B**) Heteroplasmy rate measured by restriction fragment length polymorphism (RFLP). The 3243A>G mutation was digested by Apa1, while the 10158T>C mutation was not digested by Taq1. MELAS: Mitochondrial myopathy, encephalopathy, lactic acidosis, and stroke-like episodes.

Fibroblasts were cultured from skin biopsy samples obtained from two patients with heteroplasmic mtDNA mutation and two wildtype control individuals (Figure 1A). To determine the proportion of mutation in cells, polymerase chain reaction restriction fragment length polymorphism (PCR-RFLP) analysis was performed (Figure 1B). In the results obtained by PCR-RFLP, as expected, control fibroblasts had no abnormalities, and MELAS patient fibroblasts and Leigh patient fibroblasts confirmed mtDNA mutations to some extent.

### 3.2. Oxygen Consumption Rate in MD Fibroblasts

**Figure 2:**
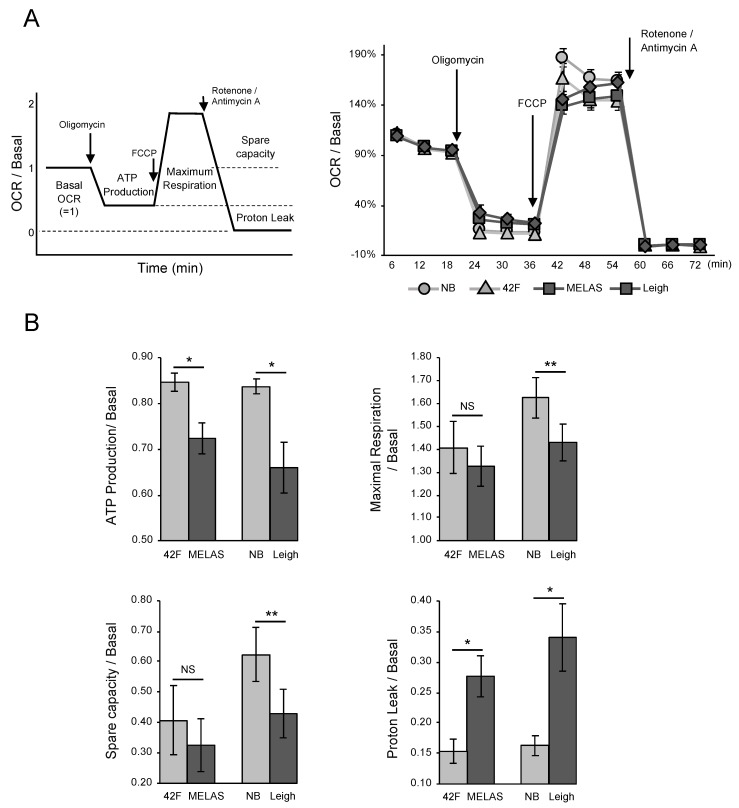
(**A**) Oxygen consumption rate (OCR) in healthy fibroblasts and mitochondrial disease (MD) fibroblasts. Mitochondrial respiration, reflected by OCR levels, was detected following the addition of oligomycin, carbonyl cyanide-p-trifluoromethoxyphenyl-hydrazon (FCCP), the uncoupler, or the electron transport inhibitor rotenone and antimycin A. Actual measurement values were normalized with basal respiration. The left panel shows a schematic of the experiment on OCR. (**B**) Opposite to the decrease in adenosine triphosphate (ATP)-linked OCR, proton leak increased in MD fibroblasts. Rates of ATP production, maximal respiration, spare capacity, and proton leak were quantified by normalization of basal OCR. The above data were graphed for each item. Error bars represent ± SD (*n* = 4 independent experiments). *P < 0.05, **P < 0.01 (Student’s t-test).

Next, we determined mitochondrial respiration by measuring the oxygen consumption rate (OCR) in control and MD fibroblasts. The bioenergetic status of different control and patient fibroblasts was assessed by monitoring oxygen consumption in a Seahorse Bioscience XFp extracellular flux analyzer (Figure 2A). In the experiment, the analysis was performed with pairs close to the patient’s age (MELAS sample vs. sample from a healthy individual aged 42 years, Leigh sample vs. sample from a healthy newborn). Basal respiration, maximal respiration, adenosine triphosphate (ATP)-linked OCR, and proton leak-linked OCR were analyzed in fibroblasts from two control individuals and two MD patients (Figure 2A). Although tendencies toward decreased ATP-linked OCR and increased proton leak were observed, reserve capacity and maximal OCR were not significantly changed in MELAS fibroblasts. In Leigh fibroblasts, basal, maximal, and ATP-linked OCR were decreased, and proton leak-linked OCR was increased (Figure 2B). Unlike the decrease in ATP-linked OCR, the proton leak was increased in the two MD fibroblast samples. These data indicate that the MD fibroblast mitochondria were dysfunctional and, as a result, were unable to use oxygen efficiently.

### 3.3. Mitochondrial Membrane Potential and ATP Production

**Figure 3:**
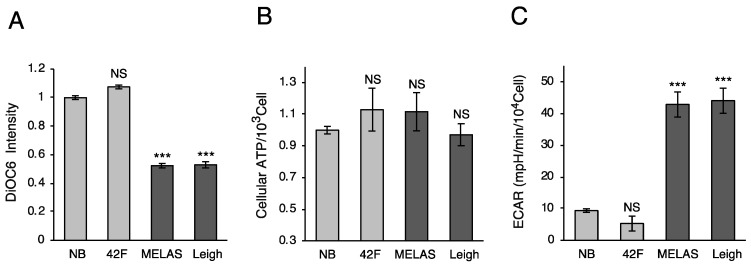
Mitochondrial functions in MD fibroblasts. (**A**) Mitochondrial membrane potential is reduced in MD fibroblasts. Mitochondrial membrane potential was measured by staining the cells with 3,3′-dihexyloxacarbocyanine iodide (DiOC6) fluorescent dye and subsequently analyzed using flow cytometry. (**B**) ATP levels are not different between MD and healthy fibroblasts. Cellular ATP concentrations were assessed using CellTiter-Glo® (Promega). (**C**) Increased glycolysis in MD fibroblasts. Extracellular acidification rate (ECAR) was measured in MD and healthy fibroblasts by using Seahorse xFp. All data were analyzed using one-way ANOVA followed by Dunnett’s test. Error bars represent ± SD (*n* = 4 independent experiments). ***P < 0.001 compared with new born (NB).

To observe mitochondrial function specifically, we tried to measure mitochondrial membrane potential (ΔΨ) and ATP content. Membrane potential changes were measured in MD fibroblasts by using the carbocyanine dye 3,3′-dihexyloxacarbocyanine iodide (DiOC6(3)). Both cell lines showed a significant reduction in ΔΨ compared with control fibroblasts (Figure 3A). Additionally, we measured the ATP content luminometrically in MD fibroblasts. The ATP content did not change significantly, although mitochondrial function was impaired (Figure 3B). This indicated that the ATP content was complemented by glycolysis in MD fibroblasts. We tried to quantify intracellular rates of glycolysis using a Seahorse extracellular flux analyzer. Consistent with these results, glycolysis was markedly upregulated in MD fibroblasts (Figure 3C). Thus, these data indicate that mitochondrial dysfunction occurs in MD fibroblasts and that MD fibroblasts use anaerobic glycolysis.

### 3.4. Promotion of Mitochondrial Fragmentation in MD Fibroblasts

**Figure 4:**
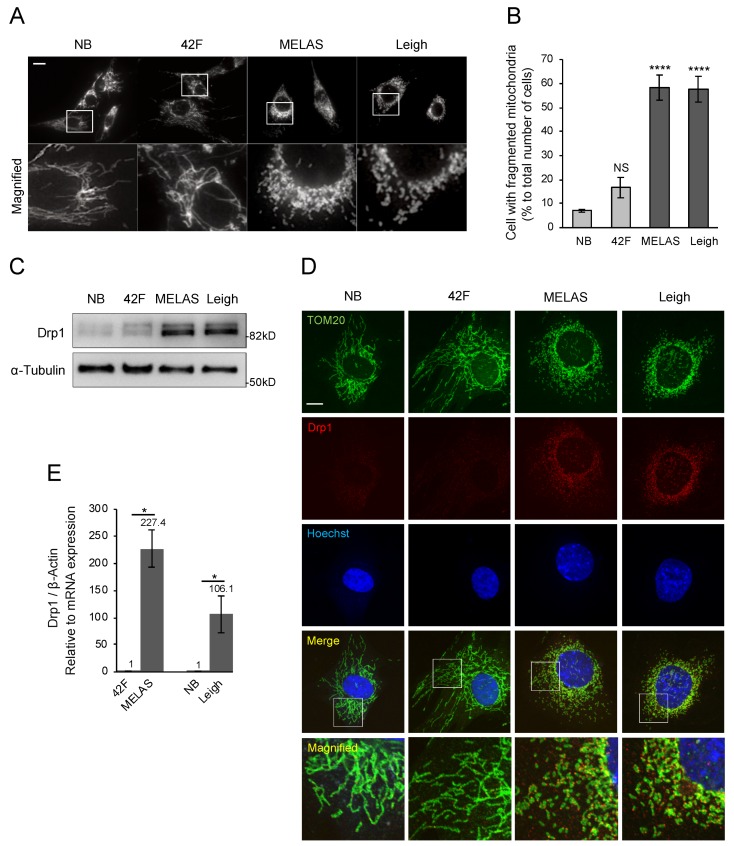
Mitochondrial fragmentation in MD fibroblasts. (**A**,**B**) Mitochondrial fragmentation is increased in MD fibroblasts. Cells were stained with anti-Tom20 antibody. Scale bar represents 20 μm. Fragmented mitochondria are defined in Supplementary Figure S1A. Quantification was performed from 300 cells per experiment examined in three separate experiments. Data are shown as the mean ± SEM. Statistical analysis was performed with one-way ANOVA followed by Dunnett’s test. ****P < 0.0001 compared with NB. (**C**) Mitochondrial morphology-related proteins were analyzed by Western blotting. MD fibroblasts had increased Drp1, and alpha-tubulin was used as loading control. (**D**) Anti-Tom20 and anti-Drp1 antibodies were used to visualize mitochondria and Drp1. Drp1 accumulates on mitochondria in MD fibroblasts. Scale bar represents 10 μm. (**E**) Drp1 expression levels were analyzed by qRT-PCR and compared to age-related controls (*n* = 3 independent experiments). *P < 0.05 (Student’s t-test).

Previous studies suggested that several mitochondrial functions can be controlled by mitochondrial morphology [31,32]. However, it is not clear how mitochondrial morphology contributes to MD. To examine it in two MD fibroblasts, we first observed mitochondrial morphology by immunofluorescence using an anti-Tom20 antibody. Fragmented mitochondria are defined in Supplementary Figure S1A. Our data show that mitochondria were severely fragmented in MD fibroblasts, in contrast to the elongated mitochondria in control fibroblasts (Figure 4A,B). Mitochondrial fission depends on recruitment of Drp1 to mitochondria. We next measured intracellular Drp1 and found that it was significantly increased in MD fibroblasts compared with control fibroblasts (Figure 4C). Thus, these data indicate that mitochondria were fragmented by the mitochondrial accumulation of Drp1 in MD fibroblasts. Mitochondrial morphology is also regulated by other fission/fusion factors. We observed significant changes of several regulators of mitochondrial morphology (Supplementary Figure S1B), suggesting dramatic changes in mitochondrial morphology in MD fibroblasts. Next, we performed immunostaining to examine whether Drp1 was localized on mitochondria. Our data show that endogenous Drp1 was localized to mitochondria in MD fibroblasts, which is also consistent with a role in mitochondrial division (Figure 4D). To investigate the mechanism of Drp1 increase in MD fibroblasts, Drp1 expression levels were analyzed with real-time polymerase chain reaction and compared with Drp1 levels in control fibroblasts. Surprisingly, Drp1 showed a 200-fold increase in MELAS fibroblasts and a 100-fold increase in Leigh fibroblasts (Figure 4E), suggesting that the Drp1 protein level was increased by transcriptional regulation in MD fibroblasts.

### 3.5. Inhibition of Mitochondrial Fragmentation Enhances Cellular Toxicity and Cell Death in MD Fibroblasts

**Figure 5:**
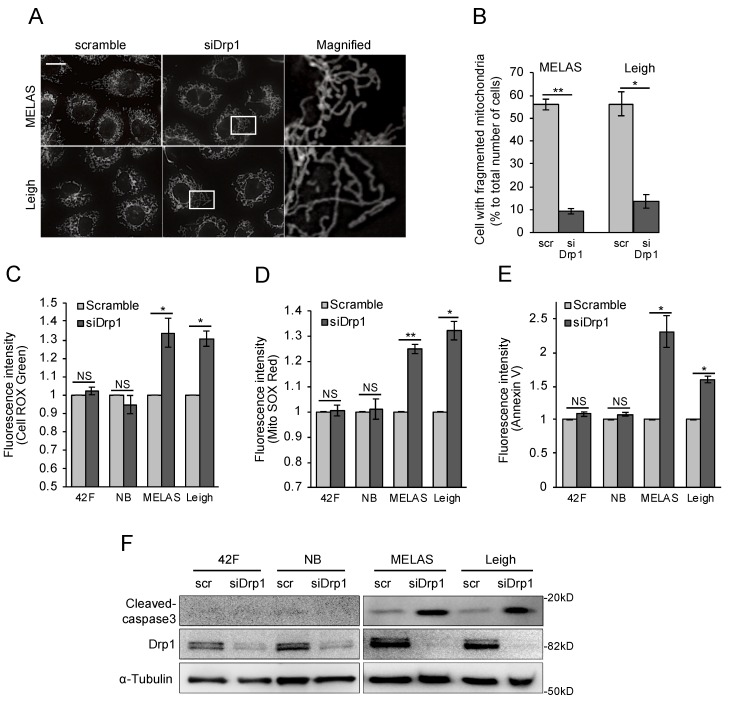
Inhibition of mitochondrial fragmentation triggers cell death in MD fibroblasts. (**A**,**B**) Drp1 knockdown approximately rescued mitochondrial fragmentation in MD fibroblasts. To visualize mitochondria, cells were stained with anti-Tom20 antibody. Scale bar represents 20 μm. Quantification was performed from 300 cells per experiment examined in three separate experiments. Error bars represent ± SD. *P < 0.05, **P < 0.01 (Student’s t-test). (**C**,**D**) Inhibition of mitochondrial fragmentation promotes intracellular reactive oxygen species (ROS) production in MD fibroblasts. MD fibroblasts were transfected with scrambled and Drp1 small interfering RNA (siRNA) for two days. To analyze oxidative stress, cells were stained with CellROX Green and MitoSOX Red and then analyzed by flow cytometry. Error bars represent ± SD (*n* = 3 independent experiments). *P < 0.05 (Student’s t-test). (**E**) Inhibition of mitochondrial fragmentation promotes cell death in MD fibroblasts. Stained with Annexin V-FITC for detection of apoptosis using flow cytometry. Error bars represent ± SD (*n* = 3 independent experiments). *P < 0.05, **P < 0.01 (Student’s t-test). (**F**) Immunoblot shows levels of cleaved caspase-3 in MD fibroblasts transfected or not with Drp1 siRNA for two days.

To examine whether mitochondrial fragmentation was caused by increased fission regulated by Drp1, experiments were performed with Drp1 small interfering RNA (siRNA). Drp1 knockdown fully rescued mitochondrial fragmentation (Figure 5A,B). To explain the action of mitochondrial fragmentation in the damage to MD fibroblasts, intracellular and mitochondrial reactive oxygen species (ROS) levels were measured in MD fibroblasts after treatment with siRNA specific for Drp1. Interestingly, intracellular ROS levels were prominently increased in MD fibroblasts after inhibition of mitochondrial fragmentation (Figure 5C,D). The presence of excess cellular levels of ROS causes cell death. Due to excess ROS release in MD fibroblasts, we used flow cytometry with annexin V to check whether blocking mitochondrial fission would accelerate cell death. Blocking of mitochondrial fission induced cell death in MD fibroblasts, linking the morphogenic machinery of this organelle to cell death induction (Figure 5E). Caspase-3 is activated by the upstream caspase-8 and caspase-9, and because it serves as a convergence point for various signaling pathways, it is well suited as a readout in apoptosis assays. We observed a significant increase in the level of active caspase-3 in MD fibroblasts coordinated with an increase in cellular ROS (Figure 5F). Thus, we found that inhibition of mitochondrial fragmentation was harmful to MD fibroblasts.

## 4. Discussion

MD refers to a group of disorders caused by mitochondrial dysfunction. High ROS is the main pathogenic factor that causes most clinical manifestations of MD [33]. Mitochondrial fission and fusion play critical roles in maintaining functional mitochondria. It has been reported that mitochondrial fission is associated with pathological conditions and mitochondrial dysfunction [34]. However, mitochondrial morphology in MD has been unclear. Here, we have shown that mitochondria were severely fragmented in MD (3243A>G, 10158T>C mutation) fibroblasts. An increase in the expression of the division factor Drp1 was also observed in MD fibroblasts, in keeping with the results of mitochondrial fragmentation. Previous studies suggested that abnormal mitochondrial fragmentation is the key factor in aging and in several diseases [35]. Therefore, inhibitors of mitochondrial fission are expected to be good therapeutic targets for diseases. Previous studies identified several mitochondrial fission inhibitors: Mdivi [36], P110 [37], and DDQ [38]. In this study, our data show that mitochondria were severely fragmented in MD fibroblasts and that inhibition of mitochondrial fragmentation by siDrp1 enhanced cellular toxicity and cell death in MD fibroblasts. Thus, our results suggest that in some situations, inhibition of mitochondrial fragmentation should be carefully controlled as a therapeutic target for mitochondrial disease. However, our results were obtained from cells of two patients with mitochondrial disease. Mitochondrial diseases can be caused by various mutations and polymorphisms in both the mitochondrial and nuclear genomes. Therefore, considering the variability of mitochondrial disease, it is important to examine whether other mutations induce mitochondrial morphology. A larger study will be necessary to understand the comprehensive mechanisms of mitochondrial disease development.

The mitochondrial network fragments into many small, round mitochondria upon entry into the stationary phase [39,40,41], and fragmented mitochondria are found in quiescent and respiratory inactive cells [30]. In the present study, two MD patient fibroblasts disrupted mitochondria dynamics, especially fission, and confirmed excessive production of reactive oxygen species induced by mitochondrial elongation via Drp1 knockdown. Therefore, we consider that mitochondrial fragmentation keeps low metabolism levels to reduce cytotoxicity in MD fibroblasts. Mitochondrial fission promotes clearance of damaged mitochondria through a form of autophagy known as mitophagy [42], whereas perturbation of mitochondrial dynamics has been shown to cause lysosomal dysfunction and autophagy impairment [43], and chronic mitochondrial defects trigger general impairment of autophagy and lysosomes to avoid complete degradation of the mitochondrial network [44,45]. Drp1 knockdown or treatment with bafilomycin A1, an autophagic flux inhibitor, significantly increased the amount of mitochondria in MD fibroblasts (Supplementary Figure S2A–C). Based on these data, MD fibroblasts lead to mitochondrial fragmentation to smoothly induce autophagy, and partly mitophagy, for reduction of cytotoxicity. Therefore, mitochondrial fission has two aspects: The beneficial function of promoting effective mitophagy and the detrimental function of releasing cytochrome c from mitochondria, which activates apoptosis signaling. In the treatment of MD, previous studies showed that induction of starvation signals by mTOR inhibition improved the pathology in the mouse model [46], and that mitochondrial division regulated the heteroplasmy rate [47]. Furthermore, starvation signals increased macroautophagy and rescued many of the metabolomic defects. It is thought that mitochondrial division effectively induces autophagy and mitochondrial fission may enable more effective treatment for MD.

## 5. Conclusions

In this study, we investigated the role of mitochondrial morphology in MD. We provide evidence that mitochondrial morphology is altered in MD fibroblasts because of the high expression level of the fission protein Drp1. Fragmented mitochondria are frequently found in MD fibroblasts, but inhibition of mitochondrial fission enhances cellular toxicity and cell death in MD fibroblasts, suggesting that mitochondrial fission plays an important role in attenuation of mitochondrial damage in MD fibroblasts.

## Supplementary Materials

The following are available online at https://www.mdpi.com/2218-273X/10/3/450/s1, Supplementary Figure S1. Mitochondrial morphology and mitochondrial morphology-related proteins; Supplementary Figure S2. Inhibition of mitochondrial fragmentation in MD fibroblasts accumulates dysfunctional mitochondria.
