# Supplementary material for: Mitochondrial Dynamics Regulation in Skin Fibroblasts from Mitochondrial Disease Patients
Source: Biomolecules. 2020 Mar 13;10(3):0. doi: 10.3390/biom10030450 (PMC7175126; doi:10.3390/biom10030450)
Supplement: Supplementary file 1 [file biomolecules-10-00450-s001.pdf]

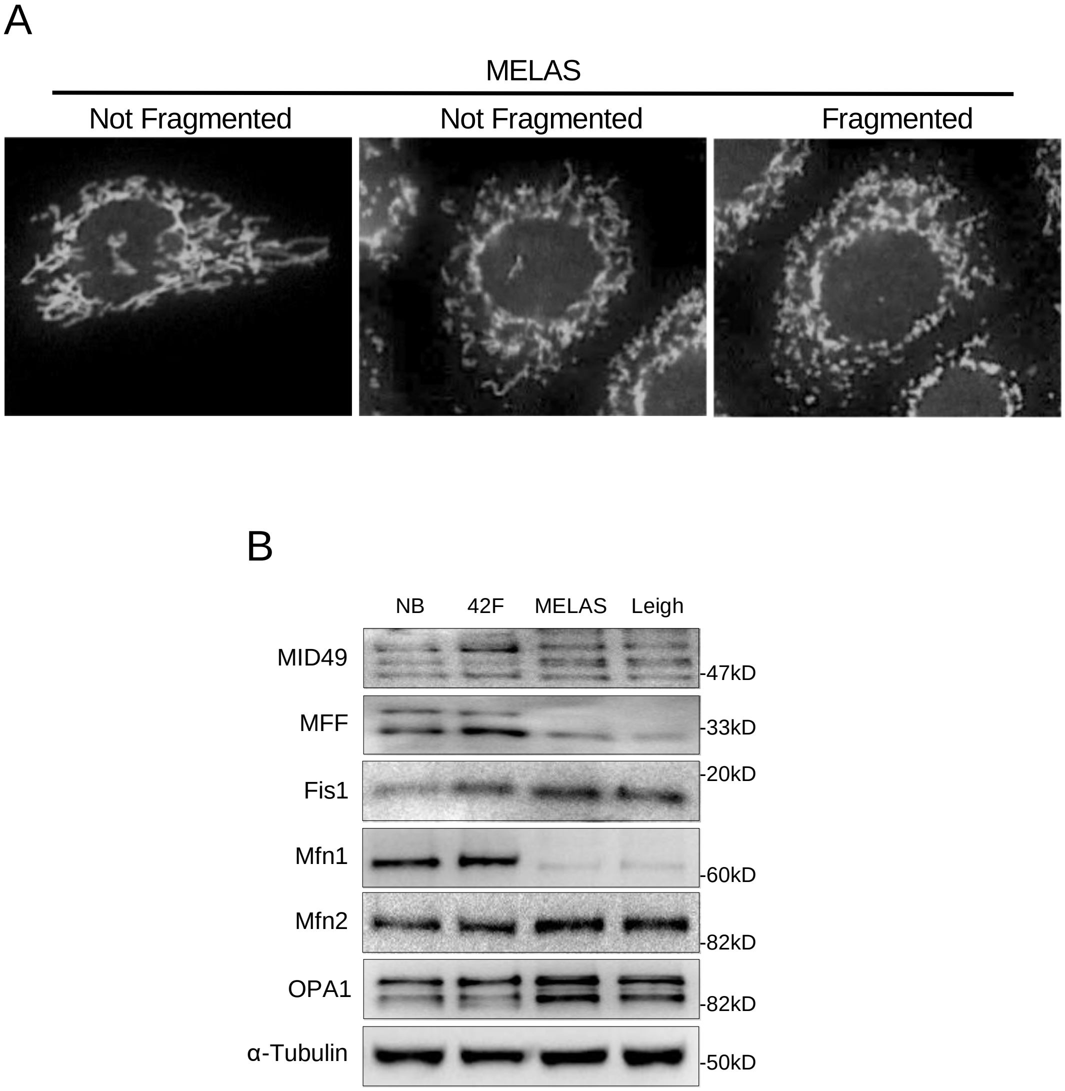

Supplementary Figure S1. (**A**) The mitochondrial morphology was defined as in images. Staining was performed with Anti-Tom20 to visualize mitochondria. (**B**) Mitochondrial morphology-related proteins ware analyzed by Western blotting. MID49, MFF, and Fis1 detected as Drp1 receptors. Mfn1, Mfn2 and Opa1 were confirmed as mitochondrial fusion factors.

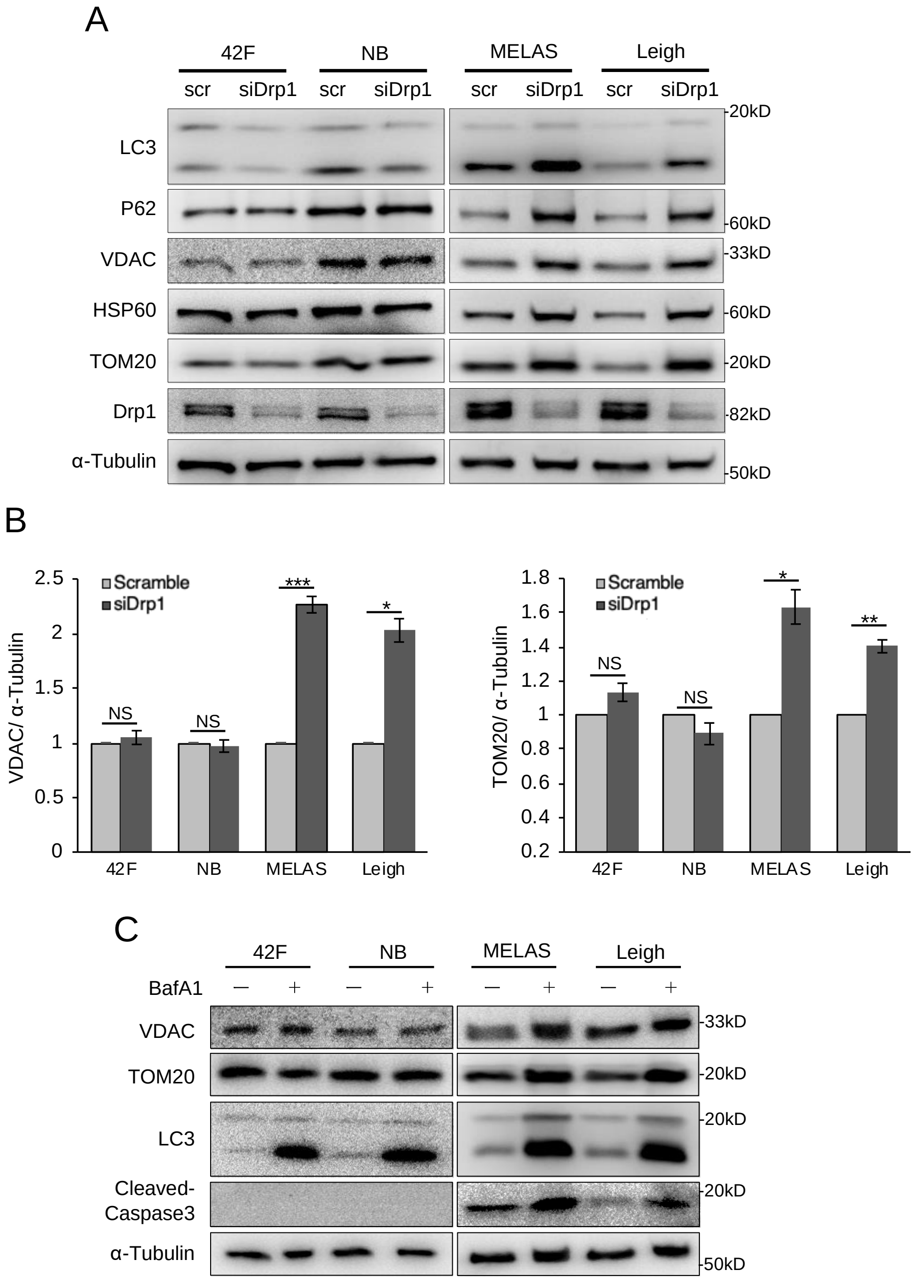

Supplementary Figure S2. Inhibition of mitochondrial fragmentation in MD fibroblasts accumulates dysfunctional mitochondria. (**A**) Inhibition of mitochondrial fragmentation increases amount of mitochondria in MD fibroblasts. Fibroblasts were transfected with scrambled and Drp1 siRNAs for 2 days. The amount of mitochondria were analyzed by Western blotting. (**B**) Quantification of amount of mitochondria (*n* = 3 independent experiments). Error bars represent ±SD. $^{*}P < 0.05$, $^{**}P < 0.01$ (Student's t-test). (**C**) Lysosomal inhibition increases amount of mitochondria and cell death in MD fibroblasts. Western blot analysis of amount of mitochondria with stimulated DMSO (control) or bafilomycin A1 (BafA1, 100 nM, 24 h).
